# Extended-Family Talk about Sex and Teen Sexual Behavior

**DOI:** 10.3390/ijerph16030480

**Published:** 2019-02-06

**Authors:** Jennifer M. Grossman, Alicia D. Lynch, Amanda M. Richer, Lisette M. DeSouza, Ineke Ceder

**Affiliations:** 1 Wellesley College, 106 Central Street, Wellesley, MA 02481, USA; aricher@wellesley.edu (A.M.R.); ldesouza@wellesley.edu (L.M.D.); iceder@gmail.com (I.C.); 2 Lynch Research Associates, 1 South Avenue, Natick, MA 01760, USA; aliciadlynch@gmail.com

**Keywords:** adolescent reproductive health, family communication, extended family, teen sexual behavior

## Abstract

Research shows that family communication about sexuality can protect against teens’ risky sexual behavior. However, few studies assess talk with extended family about sex or how this communication relates to teens’ sexual behavior. The current study includes cross-sectional survey data from 952 adolescents. Structural equation modeling (SEM) was used to assess associations between teens’ sexual risk behaviors and communication with extended family about protection methods, risks of sex and relational approaches to sex, defined as talk about sex within a close relationship. For sexually active teens, talk about protection methods was associated with fewer sexual partners and talk about risks of sex was associated with more sexual partners regardless of teen gender and the generation of extended family with whom teens talk. Results suggest that extended-family talk about sex may influence teens’ sexual behavior independent of effects of teen–parent communication. However, the direction of the effect depends on the content of the conversations. These findings suggest the need to explore whether and how extended family could be included in health prevention and intervention programs, because programs which include family largely focus on parents.

## 1. Introduction

Risky sexual behaviors, such as early onset of sexual activity and lack of protection, leave teens vulnerable to sexually transmitted infections (STIs) and unplanned pregnancy [1]. Research shows that family sexuality communication can protect against teens’ risky sexual behavior [2,3], but most studies on this topic focus exclusively on the parent–teen dyad [4]. This focus overlooks findings that over half of teens talk with extended family about sex or relationships [5]. Preliminary quantitative research shows significant associations between extended-family sexuality communication and teen sexual beliefs and behavior [5,6], but existing research often uses a single item to assess extended-family sexuality communication [7,8] and does not examine under what conditions extended-family sexuality communication can be protective or risk-promoting.

Dittus’ conceptual model posits that associations between parent sexuality communication and teens’ sexual behavior are shaped by teens’ gender and their satisfaction with their maternal relationships [9]. Studies support associations between parent–teen sexuality communication and teen sexual behavior. These studies primarily focus on mother–teen communication [4,10] and suggest that teen gender can shape these associations [4,11], although prior studies have not shown evidence for the moderating role of teen–parent relationships, such as closeness or relationship satisfaction. Research is needed to assess whether this model extends to adolescents’ sexuality communication with extended family.

Extended-family sexuality communication may be particularly relevant for Black and Latinx teens. First, these groups show high levels of communication about sex with extended family [12] and demonstrate extended family as playing a central role in teens’ development [13,14,15]. Second, Black and Latinx teens shower high rates of teen pregnancy compared to White teens [16]. Therefore, Black and Latinx teens were the primary focus for recruitment for this sample.

Communication with extended family may be particularly important as teens become sexually active. At this stage, teens may become reluctant to talk with their parents about sexual issues as they fear their parents might judge them or worry about their sexual behaviors [17,18]. Hence, some teens seek out extended family as a more comfortable alternative to parents [19]. In addition, parents often focus their messages to teens on delaying sex [20] which can be protective before teens become sexually active [21] but may be less effective when teens have already had sex. Extended family may be more open to discussing protection methods with teens, rather than only focusing on messages to delay sex [20]. Therefore, it is important to investigate extended-family sexuality communication and teens’ safer sex practices (e.g., condom use, number of partners) among teens who are sexually active, rather than a sole focus on delaying sex.

Whether messages about sex are protective may depend on which extended family member the teen talks to. For example, findings are mixed as to whether older siblings positively or negatively influence younger siblings’ sexual behavior [22,23,24], while talking with grandmothers about sex and relationships has shown associations with healthy teen sexual behavior [6,25]. These findings suggest that the generation of family that teens talk to may shape whether sexual messages are risk- or health-promoting for teens’ sexual behavior.

While who a teen talks to can shape whether communication is protective or risk-inducing, a teen’s gender, closeness with the extended family member, and level of familism can shape the strength of these associations. Familism is distinct from closeness in that it refers to a cultural value of family relationships, while closeness refers to how connected a teen feels to a specific family member. Findings are mixed regarding whether the impact of family sexuality communication differs for girls and boys [4,11,26]. These inconsistent findings may relate to differences in messages across teen gender, which are more focused on abstinence and delay of sex for girls than boys [27,28], as well as parents’ greater frequency of talk about sex and relationships with their daughters than their sons [27]. Moderating effects of teen gender between extended-family sexuality communication and teens’ sexual behavior have not been explored.

Research shows that the quality of parent–teen relationships is associated with teens’ sexual health outcomes, such as the number of sexual partners and use of birth control [29,30,31]. While closeness with extended family has not been assessed for teen sexual behaviors, close relationships with extended family are associated with positive teen health outcomes, such as adjustment [32]. Familism entails a cultural belief in the importance of family interdependence [13]. The value of familism is particularly relevant in Latino and Black cultures, where extended family provides critical support for childrearing [14,33]. A teen who places a high cultural value on family relationships and has a close connection with the extended family member he or she talks to may be more likely to listen to and adopt the family member’s beliefs about sex.

The current study extended current research by assessing (1) associations of extended-family sexuality communication with teens’ sexual behaviors; and (2) whether the links between direct communication and teen sex outcomes differ based on generation of extended family, teen gender, closeness, and familism. We hypothesized that extended-family communication about protection and relational communication about sex (sex in the context of a close relationship) would be positively associated with participants’ safer sexual behaviors, while communication about the risks of sex (e.g., pregnancy, sexually transmitted infections) would be associated with participants’ less safe sexual behaviors. We had no expectations for extended-family sexuality communication to be associated with whether teens have had vaginal sex. We expected stronger associations between extended-family sexuality communication for teens who report higher familism and more closeness with extended family. Analyses assessing whether effects of communication differ by extended-family generation and teen gender were exploratory.

## 2. Materials and Methods

### 2.1. Recruitment and Participants

Data for this study came from 11th and 12th grade students at six urban schools. Schools were recruited through school and district offices. Each school assigned a study liaison responsible for data collection coordination and was paid a $500 stipend for participation. All participants gave their informed consent for inclusion before they participated in the study. The study was conducted in accordance with the Declaration of Helsinki, and the protocol was approved by the Wellesley College Institutional Review Board (19 December 2016). An online survey was developed and field-tested with 10 high school students in a pilot phase. In this field test, students took the survey then gave verbal feedback about their perceptions of the survey. Adjustments were made to the survey based on pilot feedback. The final survey had a 6th grade reading level, established by the Flesch–Kincaid Grade Level readability test. The survey was administered using Qualtrics and was available in English and Spanish. Fifty-eight students filled out the survey in Spanish. 

In all schools, parents were sent information about the study translated into families’ home languages. Schools determined whether to use active consent or allow for a waiver of documentation of consent (“passive consent”). Four of the six participating schools selected waiver of documentation of parental consent (“passive consent”), while two schools selected active consent.

In each participating school, the liaison worked with the research team to plan data collection procedures. Teachers and administrators in the schools did not have access to any student responses but were present in the classrooms during survey administration. A total of 967 surveys were collected. Fifteen surveys were excluded from analysis because students agreed to participate but did not answer any of the survey questions. The remaining 952 participants were included in study analyses. Participants were 952 adolescents (M_age_ = 17.02, SD = 0.93), and self-identified as 55% female, 54% Latinx, 17% Black, 16% White, 7% Asian, 4% Middle Eastern, and 2% Biracial/Multiracial. On average, mothers of participants (or primary caregivers) had a high school education, and 71% of them immigrated to the U.S. Eighty-seven percent of participants reported they were attracted to opposite-sex teens, while 13% of participants were attracted to same-sex teens, both males and females, neither males nor females, or were unsure. Thirty-five percent of adolescents reported having vaginal sex. Fifty percent reported talking to at least one parent about dating, sex or relationships and almost half (46%) reported talking to an extended family member about these topics. The extended family members who teens primarily reported talking to were: Older sisters (24%) and older female cousins (21%). Older brothers (16%), aunts (13%), and older male cousins (10%) were also reported as communication partners. Fewer adolescents reported talking to grandmothers (7%), uncles (6%), grandfathers (1%), and godparents (1%). 

### 2.2. Measures

We conceptualized extended family to focus on nonparental familial relationships, including aunts and uncles, grandparents, older cousins and older siblings, and godparents. These family members may live with the teen or in a different household. We included siblings because research suggests commonalities between teens’ sexuality communication with siblings and cousins [12,19].

Questions about direct communication with extended family asked participants to focus on the extended family member “who talks to you the most about dating or sex.” Direct communication was measured using three latent variables that assessed different types of communication about sex: Protection communication, risk communication, and relational communication. These scales were adapted from the parent–adolescent communication scale (PACS) [34] and a measure of parent–child communication about sex-related topics [35]. All three scales use the same response format, which asked youth to respond to each item using a 5-point Likert scale ranging from 1 (“Never”) to 5 (“All the time”) to indicate how frequently his/her extended family member uses this type of communication. Protection Communication was measured as a latent variable comprising three indicators asking whether extended family members talk to teens about protecting themselves from sexually transmitted infections (STIs), HIV/AIDS, and from becoming pregnant or getting someone else pregnant (α = 0.93). Risk Communication was measured as a latent variable comprising four items asking whether extended family members talk to teens about the negative consequences of sex, including teen pregnancy and STIs. (α = 0.86). Relational Communication was measured as a latent variable comprising three items addressing whether extended family members talk about sex being permissible in the context of a relationship (α = 0.87). 

To create a measure representing the generation of the extended family members, a three-category variable was created. The first category included individuals who were the same generation of the youth respondents, such as older siblings and cousins. The second category consisted of family members who were likely to be the same generation as the youth’s parents, such as aunts/uncles and godparents. The third category included grandparents. 

*Teens’ closeness with their extended family member* was assessed with a single item which used a scale ranging from 1 (“Not at all close”) to 5 (“Very close”) for teens to report how close they felt to their extended family member. 

Teens’ familism values were measured using a four-item scale (α = 0.89) adapted from a familial interconnection scale [36], asking teens to describe their views on the importance of relationships with extended family members. Teens used a 5-point Likert scale ranging from 1 (“Strongly disagree”) to 5 (“Strongly agree”) to respond to items such as “A person should cherish the time spent with their relatives.”

To reduce the possibility of omitted variable bias, a series of control variables were included in each analytical model. Teens’ gender was coded as male (0) or female (1). A small number of youths who selected a non-binary gender were coded as missing. Youth age ranged from 14–21 and was measured continuously.

Youth race/ethnicity was assessed by presenting youth with a list of eight racial/ethnicity categories and asking youth to select the category or categories that best described them. Youth who selected multiple categories were coded as “multiracial”. The only race/ethnicity groups with sufficient power to warrant being modeled separately were Black, Latinx, White, and Asian. Because a statistically valid interpretation of results generated from the smaller racial/ethnic groups was not possible due to the small sample sizes, all other individuals were grouped into a category labelled “Other”. 

Household structure was a dichotomous measure coded as “1” if the youth reported their primary residence comprised two parental figures and “0” if the youth reported not living with two parental figures. Household structure options included two parents living in the same place, two parents living in different places, mother only, father only, other relatives, and people outside the family. Teens’ religiosity was measured using a single item that asked teens to use a scale ranging from 1 (“Very unimportant”) to 4 (“Very important”) to describe how important religion is in their life. Teens also reported their mothers’ education, whether their parents were immigrants, and whether either of their parents was a teen parent. A dichotomous indicator was coded as “1” if the teen self-reported talking with at least one parent about dating or sex and “0” if the teen indicated they did not talk to a parent about dating or sex.

Sexual behavior outcomes included vaginal sex, number of sexual partners, and condom use. Vaginal sex was measured dichotomously as “1” if the youth had engaged in vaginal sex or “0” if the youth had never had vaginal sex. Youth reported the number of vaginal sex partners using a six-point response scale where they indicated whether they had 1, 2, 3, 4, 5 or 6 or more vaginal sex partners in the past 12 months. Youth who reported they had not had sex in the past 12 months were given scores of “0”. Condom use was assessed using a 5-point Likert scale ranging from 1 (“Never”) to 5 (“Always”), where youth indicated how often they had used condoms during vaginal sex in the past 12 months. 

### 2.3. Analysis

Before testing analytical models exploring associations between extended-family sexuality communication and adolescent sexual behaviors, we conducted psychometric analyses that examined the proposed structure of the three direct communication scales (protection, risk, and relational communication). Model fit statistics from a confirmatory factor analysis (CFA) testing the presence of three direct communication scales suggested an excellent fit (CFI = 0.97, TLI = 0.96, RMSEA = 0.07) for the proposed theoretical structure. Cronbach’s alphas of the resulting scales further suggested excellent reliability (protection α = 0.92, risk α = 0.85, and relational communication α = 0.87).

Following the psychometric modeling of the direct communication scales, analytical models examining the primary research questions were pursued. First, separate structural equation models (SEM) were used to test associations among extended-family direct sexuality communication and each of the three youth sexual behavior outcomes (vaginal sex, number of partners, and condom use). Next, a series of interaction models examined whether the link between extended-family sexuality communication and teen sexual behavior outcomes varied in relation to the generation of the extended family member, teen gender, closeness with extended family member, and teen familism values. 

**Figure 1:**
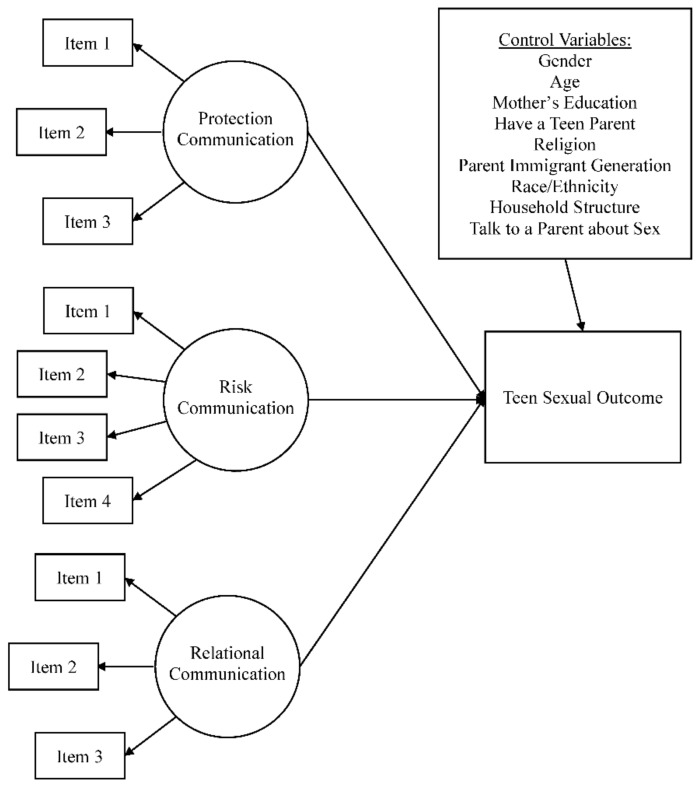
Structural equation measurement model of communication and sexual outcomes.

Each SEM comprised a measurement model and a structural model (described previously and listed in Figure 1 below). The measurement model contained latent variables representing each of the three direct communication scales (protection, risk, and relational communication). In the structural model, each of the observed exogenous variables and the three latent variables representing direct sexuality communication were regressed on the youth sexual behavior outcome. In the moderation models, additional latent variables representing each moderating effect were also regressed on the youth sexual behavior outcome. The control variables (described previously in the measures section) were included in all models. 

Given that vaginal sex was measured dichotomously, model parameters were generated using a diagonally weighted least squares estimator (WLSMV) and pairwise estimation of missing data. For the models predicting youths’ number of sexual partners and condom use, the outcomes were treated as normally distributed and model parameters were estimated using full information maximum likelihood (FIML). All analyses were conducted in the lavaan package (version 0.6-3) in R [20].

## 3. Results

Model fit statistics for the three models assessing direct effects of extended-family direct communication and teens’ vaginal sex, number of sex partners, and condom use suggested excellent fit. Specifically, the Tucker–Lewis Indices (TLI) ranged from 0.95–0.98, comparative fit indices (CFI) from 0.94–0.96, and root mean square error of approximation (RMSEA) from 0.04–0.06. In these models, both protection and risk communication were found to be significant predictors of youths’ reported number of sexual partners. These effects went in opposite directions with protection communication predicting *fewer* sexual partners (β = −0.36, SE = 0.16, *p* < 0.05) and risk communication predicting *more* sexual partners (β = 0.55, SE = 0.24, *p* < 0.05). In the models predicting vaginal sex and condom use, extended-family sexuality communication was not a significant predictor of youths’ sexual behaviors (see Table 1). 

Following the testing of the direct effect models, the interaction models assessing extended family generation, teen gender, closeness of extended family member, and teen familism values as potential moderators of the link between direct communication and teen sexual behaviors were pursued. Results suggested that extended family generation, teen gender, and closeness of extended family member did not moderate the link between extended family members’ direct sexuality communication and teens’ sexual behavior outcomes (see Table 2 and Table 3).

**Figure 2:**
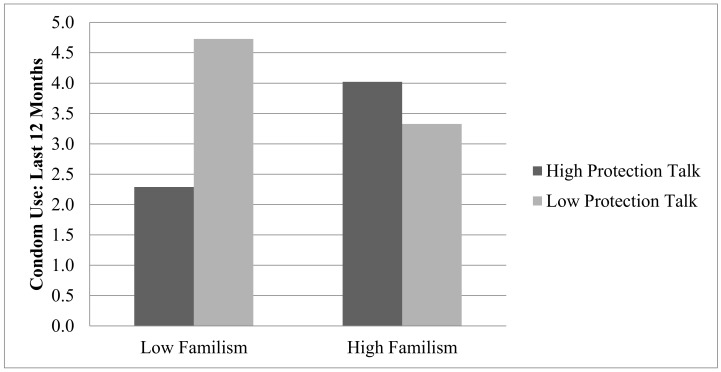
Teen familism values as a moderator of the link between extended-family protection communication and teen condom use.

However, familism values did significantly moderate the link between protection communication and teen condom use (β = 0.82, SE = 0.41, *p* < 0.05) (see Table 3). Results showed that youth with higher reported condom use had either (1) low familism values and infrequent protection communication with extended family members or (2) high familism values and frequent protection communication with extended family members. Figure 2 displays the interaction between protection communication and familism values in predicting condom use.

## 4. Discussion

Negative associations between protection communication and the number of sexual partners and positive associations between risk communication and the number of sexual partners confirm study hypotheses and suggest that Dittus and colleagues’ model [9] of parent–teen sexuality communication may apply to extended family. For sexually active teens, conversations about safer sex fit with teens’ developmental stage and experience and may encourage them to make thoughtful decisions about their sexual activity. Extended family may take on a larger role for these teens due to teens’ concerns about parent judgement regarding their sexual behavior [17] and to openness among extended family members to discussing safer sex behaviors, compared to parents, who tend to focus on delaying sex [20]. By contrast, extended-family messages about the risks of sex (reasons not to have sex) may strike a dissonant chord with teens who are already sexually active. For this group of teens, messages about delaying sex do not acknowledge their sexual behavior and suggest that sexual behavior is bad or inappropriate, while also failing to provide tools or information to support safer sex behaviors. Counter to study hypotheses, extended-family sexuality communication did not predict teens’ condom use. Future research may help to understand this unexpected finding. Relational communication was also not predictive of teen sexual risk behavior. It may be that communication needs to focus on specific safer sex practices in order to be protective. As hypothesized, extended-family sexuality communication was not associated with having vaginal sex. This may reflect parents’ key role in talking with teens about the dangers of sex, which is associated with delayed sex among teens who are not yet sexually active [37]. 

The lack of significant moderation findings for generation of extended family suggests that the protective potential of extended-family messages about sex does not depend on the age of the extended family member. This runs counter to findings that same-generation family members, such as siblings, promote risky sexual behavior among their younger teen relatives [24]. However, it fits with prior research findings that sibling communication about sex can support teens’ sexual health, especially when combined with teen–parent conversations about sex [23,38]. Together, these findings suggest that even same-age extended family should be considered by health educators as potential sources of outreach and resources to support teens’ sexual health.

Nonsignificant moderation findings for teen gender indicate the effects of extended-family messages do not differ for teen boys and girls. This finding suggests that the link between direct communication with extended family members about sex and teens’ sexual behaviors is stable regardless of teen gender. Teen gender-differences in effects of extended-family communication about sex on teens’ sexual behavior have not been assessed in past research. However, prior studies show mixed findings for the role of teen gender in relationships between parent–teen communication and teens’ sexual behavior [4,11].

The lack of moderating effects for extended-family closeness is surprising, as one would expect that teens would be more likely to listen to feedback about sex shared by family members to whom they are close. It may be that closeness is too general a construct and that being close with someone does not necessarily mean they seek them out for advice or guidance. A more specific construct related to respecting an extended family member’s opinions or listening to their advice may be more closely linked to teens’ sexual behavior.

By contrast, teens’ familism values moderated associations between extended-family talk about sex and teens’ sexual behavior. The finding for high condom use among teens with high familism values and frequent protection communication with extended family members fits with study hypotheses. This suggests that valuing family relationships in general, not just valuing one person in particular, helped to shape teens’ responses to family communication. This may tie into the notion of respect for family members. For example, familial honor and respect are often included in scales of familism, which include listening to family members’ perspectives and asking for their advice [36,39]. 

However, findings also showed that teens with low familism values and infrequent protection communication from extended family members reported high condom use, a result which is more difficult to understand. It may be that participants interpret familism values in different ways that may be more complex than what was assessed in the current study or that some teens who are more independent from family find protective resources from other sources (e.g., friends, own value system). Future research could examine a broader range of internal and external resources that may shape teens’ sexual behaviors. 

This study’s findings are limited by the cross-sectional data, which do not allow for causal inferences. Sample participants reported unexpectedly low rates of sexual behavior compared to national norms [1], which reduced the analytic power to identify relationships between direct sexuality communication and sexual behavior within the sample. Over 70% of this study’s sample self-identified as Latinx or Black, providing valuable data on groups with potential for positive health impacts from extended-family sexuality communication. However, further research is needed to assess relationships between extended-family sexuality communication and teen sexual behavior in other racial/ethnic groups. Finally, the survey definition of sex does not include nonvaginal sexual behavior and therefore does not address associations between extended-family communication and nonvaginal sex, which limits application of the study to vaginal sex without accounting for oral or anal sex. 

Future research should assess how teens’ talk with extended family about sex and their sexual behavior interact over time. Future research would benefit from longitudinal studies to assess how extended-family conversations about sex covary with the development of teens’ sexual behavior and whether the effects of this communication on teens’ sexual behavior change after teens become sexually active. This research could address questions such as whether teens are more likely to talk with extended family after they have sex, and whether this communication is more likely to protect teens from sexual risk behavior than conversations with extended family before teens become sexually active, as well as how these patterns compare with teens’ communication with their parents. Future studies could also assess constructs that more specifically assess qualities of family relationships, including whether teens listen to family members or seek them out for advice about sexual issues. 

## 5. Conclusions

Close to half of teens talk with extended family about sex [5], yet little research investigates whether and under what conditions this communication could protect teens from risky sexual behavior. While the current findings are cross-sectional, they suggest that extended-family communication may make a difference in teens’ sexual behavior. Extended family may play a particularly important role for sexually active teens, who often turn to extended family as less judgmental and more open supports for discussing sexual issues compared to parents [20]. The content of this communication is critical, as it can be either protective or risk-promoting. As shown in research with parents [40], extended family may underestimate teens sexual behavior and therefore share messages about sex that do not match teens’ behaviors and their needs for information and support. In contrast, talking with sexually active teens about how to minimize risk may support teens’ sexual health. Extended-family communication has similar effects regardless of the generation of extended family teens talk with and the gender of the teen. Overall, these findings suggest that (1) given the frequency and potential health effects of teens’ communication with extended family, these relationships should be recognized in teen health programs, which primarily focus on parents [41,42]. Counter to common fears about teens’ conversations with siblings and cousins about sex, these preliminary data suggest that programs should not discourage teens from talking with their older siblings and cousins about sex and relationships, particularly when teens do not have a parent they can talk to about these issues; (2) education is needed to support extended family members regarding which types of communication can effectively support the health of sexually active teens, as targeting communication to a teen’s development and experience is key to its protective potential. 

## Figures and Tables

**Table 1:** Direct effects of extended-family (EF) communication on teen sexual behaviors.

	Delay Sex	Condom Use	Number of Sex Partners
	β (SE)	β (SE)	β (SE)
Protection Communication	0.07 (3.92)	−0.20 (0.20)	−0.36 (0.16) *
Risk Communication	0.24 (5.93)	0.30 (0.36)	0.55 (0.24) *
Relational Communication	−0.14 (5.17)	0.17 (0.26)	−0.10 (0.17)
Gender	−0.09 (0.12)	−0.23 (0.22)	0.07 (0.16)
Age	0.16 (0.07) *	−0.19 (0.13)	0.08 (0.10)
Mother’s Education	0.04 (0.04)	−0.06 (0.07)	0.03 (0.05)
Have a Teen Parent	0.35 (0.13) **	−0.21 (0.23)	0.03 (0.17)
Religion	−0.04 (0.06)	0.09 (0.12)	−0.10 (0.08)
Parent Immigrant Generation	−0.21 (0.16)	0.04 (0.31)	0.03 (0.21)
Black (vs. White)	−0.31 (0.21)	−0.35 (0.42)	0.01 (0.31)
Latinx (vs. White)	0.13 (0.18)	−0.47 (0.34)	−0.49 (0.24)
Asian (vs. White)	−0.38 (0.27)	−0.59 (0.60)	−0.42 (0.43)
Other Race (vs. White)	−0.41 (0.3)	−0.21 (0.62)	−0.40 (0.41)
Household Structure	−0.2 (0.13)	0.04 (0.25)	−0.04 (0.17)
Talk to a Parent about Sex	0.17 (0.12)	0.43 (0.25)	0.32 (0.17)

* *p* < 0.05, ** *p* < 0.01.

**Table 2:** Teen gender and extended family generation as moderators of the relationship between extended-family (EF) communication and teen sexual behaviors.

	Teen Gender Moderation			EF Generation Moderation		
	Vaginal Sex	Condom Use	Number of Sex Partners	Vaginal Sex	Condom Use	Number of Sex Partners
	β (SE)	β (SE)	β (SE)	β (SE)	β (SE)	β (SE)
Protection Communication	0.030 (4.61)	−0.26 (0.19)	−0.35 (0.17) *	−0.05 (8.70)	0.13 (51.49)	−0.39 (0.31)
Risk Communication	0.32 (8.70)	0.36 (0.39)	0.61 (0.25) *	0.37 (46.78)	0.39 (103.27)	0.70 (0.56)
Relational Sex Communication	−0.15 (8.06)	0.15 (0.31)	−0.17 (0.18)	−0.02 (51.13)	−0.30 (67.63)	−0.05 (0.30)
Protection X Teen Gender	−0.04 (19.34)	0.55 (0.39)	−0.38 (0.34)	–	–	–
Risk X Teen Gender	−0.04 (45.31)	−0.46 (0.81)	0.16 (0.49)	–	–	–
Relational X Teen Gender	−0.16 (26.16)	0.12 (0.67)	0.12 (0.40)	–	–	–
Protection X Middle Age EF	–	–	–	0.02 (20.55)	−0.75 (25.38)	0.80 (0.71)
Risk X Middle Age EF	–	–	–	−0.05 (136.42)	0.10 (138.25)	−0.52 (1.25)
Relational X Middle Age EF	–	–	–	0.21 (125.02)	0.52 (133.22)	−0.05 (0.83)
Protection X Older Age EF	–	–	–	0.09 (177.05)	0.20 (90.03)	−1.24 (1.78)
Risk X Older Age EF	–	–	–	−0.04 (472.61)	0.03 (262.55)	3.90 (4.91)
Relational X Older Age EF	–	–	–	−0.05 (314.04)	−0.11 (171.28)	−0.22 (1.39)
Middle Age EF	–	–	–	0.12 (0.16)	−0.19 (0.25)	−0.01 (0.29)
Older Age EF	–	–	–	−0.13 (0.22)	0.66 (0.39)	−0.65 (0.35)
Gender	−0.09 (0.12)	−0.28 (0.23)	0.09 (0.16)	−0.14 (0.13)	−0.08 (0.23)	0.10 (0.19)
Age	0.16 (0.07) *	−0.18 (0.13)	0.07 (0.10)	0.11 (0.09)	0.06 (0.16)	−0.01 (0.12)
Mother’s Education	0.04 (0.04)	−0.07 (0.07)	0.03 (0.05)	0.01 (0.04)	0.00 (0.07)	0.01 (0.06)
Have a Teen Parent	0.35 (0.13) **	−0.28 (0.24)	0.04 (0.17)	0.40 (0.15) **	−0.44 (0.22) *	0.15 (0.21)
Religion	−0.04 (0.06)	0.09 (0.12)	−0.11 (0.08)	−0.05 (0.07)	−0.09 (0.13)	−0.06 (0.10)
Parent Immigrant Generation	−0.21 (0.16)	−0.01 (0.31)	0.05 (0.22)	−0.34 (0.20)	0.41 (0.31)	−0.04 (0.28)
Black (vs. White)	−0.31 (0.21)	−0.37 (0.42)	0.01 (0.31)	−0.11 (0.25)	−0.49 (0.41)	0.04 (0.38)
Latinx (vs. White)	0.13 (0.18)	−0.46 (0.34)	−0.50 (0.24) *	0.27 (0.22)	−0.54 (0.32)	−0.68 (0.31) *
Asian (vs. White)	−0.38 (0.27)	−0.55 (0.60)	−0.43 (0.43)	−0.02 (0.31)	−0.89 (0.56)	−0.51 (0.51)
Other Race (vs. White)	−0.41 (0.30)	−0.21 (0.61)	−0.40 (0.41)	−0.11 (0.34)	−0.22 (1.02)	−0.87 (0.52)
Household Structure	−0.20 (0.13)	0.07 (0.25)	−0.04 (0.17)	−0.23 (0.15)	−0.35 (0.23)	0.07 (0.21)
Talk to a Parent about Sex	0.17 (0.12)	0.48 (0.25)	0.27 (0.18)	0.26 (0.15)	0.52 (0.25) *	0.38 (0.22)

* *p* < 0.05, ** *p* < 0.01.

**Table 3:** Teen familism and teen extended family (EF) closeness as moderators of the relationship between EF communication and teen sexual behaviors.

	Teen Familism Moderation			Teen Closeness with EF Moderation		
	Vaginal Sex	Condom Use	Number of Sex Partners	Vaginal Sex	Condom Use	Number of Sex Partners
	β (SE)	β (SE)	β (SE)	β (SE)	β (SE)	B (SE)
Protection Communication	−0.05 (5.40)	−0.63 (0.28) *	−0.26 (0.20)	−0.04 (4.06)	−0.19 (0.21)	−0.36 (0.20)
Risk Communication	0.30 (7.63)	0.65 (0.40)	0.50 (0.25) *	0.63 (7.41)	0.61 (0.45)	0.53 (0.37)
Relational Communication	−0.07 (7.36)	0.25 (0.27)	−0.13 (0.19)	−0.36 (5.79)	0.13 (0.34)	0.00 (0.24)
Protection X Teen Familism	0.20 (8.61)	0.82 (0.41) *	−0.20 (0.34)	–	–	–
Risk X Teen Familism	0.10 (8.84)	−0.53 (0.62)	0.16 (0.41)	–	–	–
Relational X Teen Familism	−0.20 (11.3)	−0.35 (0.49)	0.03 (0.27)	–	–	–
Protection X Closeness with EF	–	–	–	−0.05 (4.27)	0.16 (0.22)	0.10 (0.17)
Risk X Closeness with EF	–	–	–	−0.21 (7.89)	−0.61 (0.53)	0.01 (0.39)
Relational X Closeness with EF	–	–	–	0.25 (6.62)	0.43 (0.31)	−0.16 (0.23)
Teen Familism	−0.07 (0.07)	0.27 (0.16)	−0.22 (0.11)	–	–	–
Closeness with EF	–	–	–	0.06 (0.05)	−0.19 (0.10)	−0.01 (0.08)
Gender	−0.06 (0.12)	−0.25 (0.22)	0.09 (0.16)	−0.13 (0.13)	−0.20 (0.24)	0.05 (0.19)
Age	0.18 (0.07) *	−0.21 (0.13)	0.09 (0.10)	0.11 (0.08)	−0.06 (0.16)	0.04 (0.12)
Mother’s Education	0.04 (0.04)	−0.06 (0.07)	0.03 (0.05)	0.02 (0.04)	0.04 (0.07)	−0.02 (0.06)
Have a Teen Parent	0.36 (0.13) **	−0.25 (0.23)	0.00 (0.17)	0.40 (0.15) **	−0.51 (0.26) *	0.10 (0.21)
Religion	−0.04 (0.06)	0.08 (0.12)	−0.09 (0.09)	−0.05 (0.07)	−0.18 (0.14)	−0.04 (0.10)
Parent Immigrant Generation	−0.19 (0.17)	0.14 (0.31)	−0.08 (0.22)	−0.30 (0.20)	0.41 (0.36)	0.05 (0.27)
Black (vs. White)	−0.31 (0.22)	−0.39 (0.42)	0.15 (0.32)	−0.11 (0.25)	−0.51 (0.45)	−0.02 (0.39)
Latinx (vs. White)	0.11 (0.18)	−0.56 (0.34)	−0.40 (0.25)	0.24 (0.22)	−0.66 (0.38)	−0.74 (0.31) *
Asian (vs. White)	−0.39 (0.28)	−0.69 (0.60)	−0.33 (0.43)	−0.01 (0.31)	−1.39 (0.63) *	−0.58 (0.51)
Other Race (vs. White)	−0.39 (0.31)	−0.29 (0.62)	−0.40 (0.42)	−0.15 (0.34)	−0.09 (0.64)	−0.86 (0.50)
Household Structure	−0.21 (0.13)	0.07 (0.25)	−0.03 (0.17)	−0.21 (0.15)	−0.22 (0.29)	0.02 (0.22)
Talk to a Parent about Sex	0.19 (0.12)	0.42 (0.25)	0.30 (0.18)	0.25 (0.15)	0.52 (0.28)	0.43 (0.21) *

* *p* < 0.05, ** *p* < 0.01.
